# De Winters modified electrocardiogram pattern

**DOI:** 10.1007/s12471-016-0836-4

**Published:** 2016-04-14

**Authors:** R. C. Rodrigues, S. Gomes, A. Drumond, D. Pereira

**Affiliations:** Cardiology Service, Hospital Dr. Nélio Mendonça, Funchal, Portugal

**Fig. 1:**
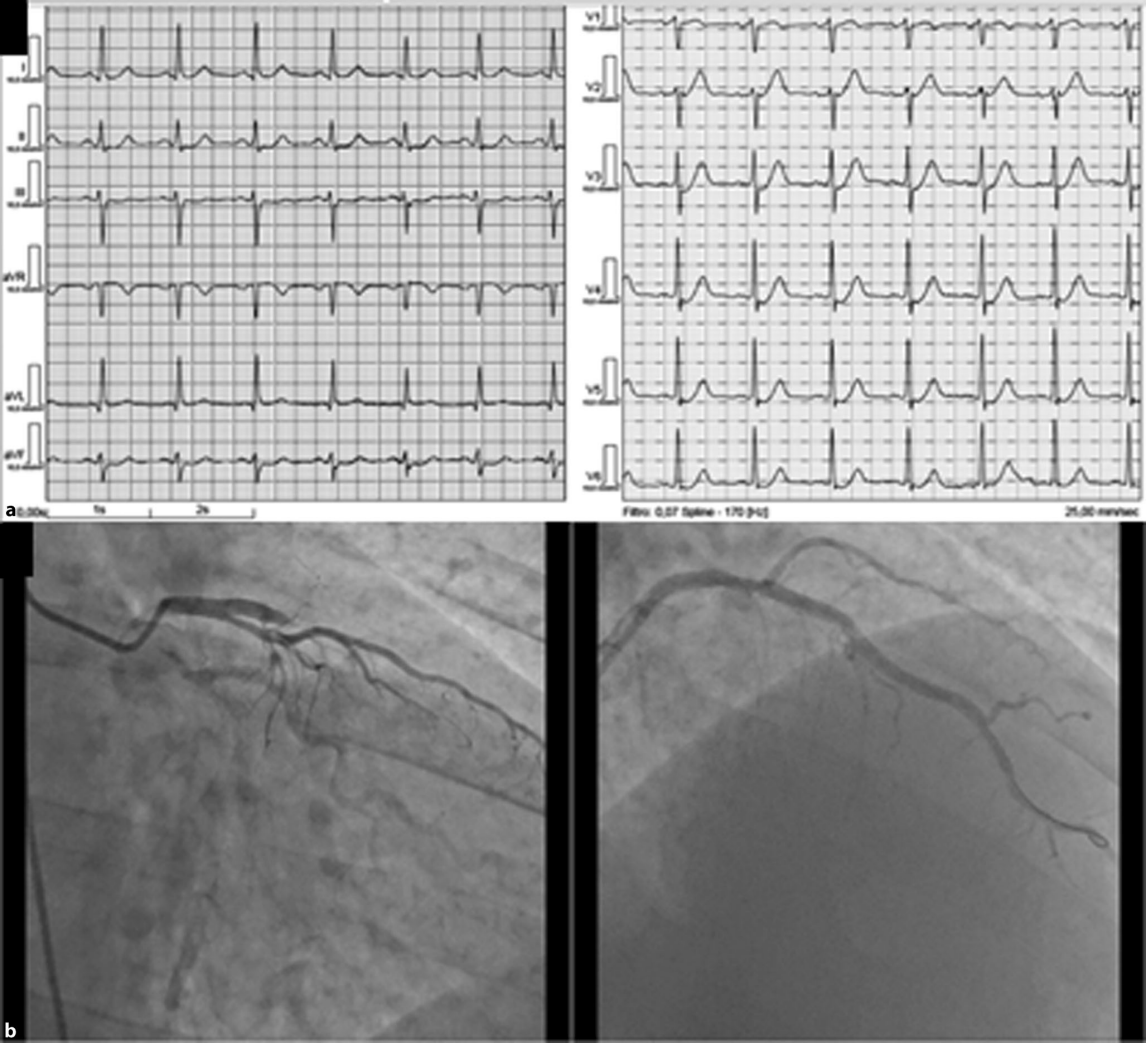
**a** 12-lead electrocardiogram showing the “modified de Winters” pattern with 1mm upsloping ST-segment depression at the J point in leads V3 to V6; **b** Angiogram showing a left kissing ostium and occlusion of proximal left anterior descending artery (*left*) and selective left anterior descending artery catheterization revealing good final result (*right*)

A 59-year-old patient with a family history of coronary artery disease and no other risk factors arrived at the emergency department with typical angina chest pain lasting for 60 minutes. The admission electrocardiogram showed 1 mm upsloping ST-segment depression at the J point in leads V3–V6 without peaked T waves (Fig. 1a). The patient underwent primary percutaneous transluminal coronary angioplasty where a kissing ostium was found and proximal anterior descending artery (LAD) occlusion was identified and stented with a drug-eluting stent (Fig. 1b), with successful reperfusion. The patient’s stay in hospital was uneventful.

This patient displayed a typical upsloping ST-segment depression but without typical peaked T‑waves or aVR ST-segment elevation, which is usually associated with a characteristic electrocardiogram pattern first described by De Winters [1] and associated to proximal LAD occlusion. The non-recognition of these unusual STEMI equivalent patterns leads to a higher reperfusion time and is associated with worse outcomes [2, 3].
